# Factors associated with anxiety, depression, and cognitive impairment in individuals with hypertension: a cross-sectional study based on a Chinese population

**DOI:** 10.3389/fcvm.2026.1888483

**Published:** 2026-08-11

**Authors:** Xinxin Zhang, Xia Zhong, Kunning Wang, Ying Shao, Zhenyuan Wang, Huachen Jiao

**Affiliations:** 1 Department of Cardiology, Feicheng City Traditional Chinese Medicine Hospital, Feicheng, Shandong, China; 2 Institute of Child and Adolescent Health, School of Public Health, Peking University, Beijing, China; 3 Medical College, Ningxia Medical University, Yinchuan, Ningxia, China; 4 Department of Traditional Chinese Medicine, Jining First People’s Hospital, Jining, Shandong, China; 5 Department of Cardiology, Shandong University of Traditional Chinese Medicine Affiliated Hospital, Jinan, Shandong, China

**Keywords:** anxiety, cognitive impairment, cross-sectional study, depression, hypertension, lifestyle

## Abstract

**Objective:**

To investigate factors associated with anxiety, depressive symptoms, and cognitive impairment in individuals with hypertension.

**Methods:**

A multicenter, large-sample cross-sectional study was conducted on 1,915 essential hypertension patients from multiple hospitals in Shandong Province (May 2022–July 2024). They were divided into four groups by Mini-Mental State Examination, Hamilton Anxiety and Depression Scales: hypertension alone (*n* = 1,350), with cognitive impairment (*n* = 191), with anxiety/depression (*n* = 309), and with all three conditions (*n* = 65). Demographics, clinical indices, lifestyle, sleep quality, and treatment information were collected. Univariate analysis, binary logistic regression, and ROC curves were used to examine these factors.

**Results:**

Independent risk factors for hypertension patients with anxiety/depression included waist circumference (OR=1.06), heart rate (1.02), education level, alcohol cessation (3.1), usual systolic blood pressure (1.02) and poor sleep; protective factors included age (0.94), height (0.96), occasional drinking (0.25), hypertension family history (0.15) and lower LDL-C (0.73). For hypertension patients with all three conditions, risk factors were waist circumference (1.07) and poor sleep. Lower LDL-C (0.62) was a protective factor. ROC curves showed good discriminative capacity (AUC=0.895, 0.887).

**Conclusion:**

Among individuals with hypertension, comorbid anxiety, depression, and cognitive impairment are associated with poorer sleep quality and elevated waist circumference. Lower LDL-C and appropriate lipid management may have protective effects.

## 1 Introduction

Hypertension ranks among the most common chronic conditions worldwide, with its incidence continuing to escalate annually (1). Elevated blood pressure, according to the 2019 Global Burden of Disease Study, is the foremost attributable risk factor for mortality worldwide, accounting for 10.8 million deaths annually. It is also the principal contributor to the burden of cardiovascular disease (2). Hypertension can disrupt cerebral microcirculation, inducing structural and functional alterations in the brain that compromise cognitive performance (3). Multiple investigations have corroborated that hypertension constitutes a major risk factor for cognitive decline and Alzheimer's disease (4–6). The prevalence of mild cognitive impairment (CI) comorbid with hypertension approximates 30% (7), a condition that markedly impedes reversion to normal cognitive status (8), thereby imposing substantial living and economic strains on affected individuals. Furthermore, inadequately controlled hypertension exerts severe somatic repercussions, precipitating disease-related apprehension and considerable psychological distress, including anxiety and depression (9).

A growing body of research has focused on the bidirectional relationship between hypertension and emotional disturbances, particularly anxiety and depression. Recent evidence indicates that anxiety and depression elevate the likelihood of hypertension in normotensive populations by 3.5- and 2.0-fold, respectively (10), with comorbidity rates being higher among younger women (11). The influence of anxiety or depression on hypertension may involve mechanisms such as activation of the autonomic nervous system, immune dysregulation, and disturbances of hormonal systems (12, 13). These emotional states attenuate cardiovascular stress reactivity, thereby augmenting the risk of cardiovascular diseases (10). A cross-sectional investigation suggests that sleep disturbance and depression might mediate the correlation of hypertension with CI in adults aged 65 and above (14). Prior work has demonstrated associations of sleep disturbance, occupational intensity, and intensity of physical activity with CI in individuals with hypertension (15, 16). Nevertheless, the majority of investigations have examined the correlation of hypertension with anxiety or depression in isolation (17). A research gap persists concerning the nexus among hypertension, comorbid anxiety and depressive symptoms, and CI. Consequently, this multicenter, large-sample cross-sectional investigation aims to delineate pivotal risk and protective factors for anxiety and depressive symptoms combined with CI in a hypertensive cohort. The findings may furnish new targets for optimizing comprehensive management strategies, refining emotional regulation, and enhancing protocols of cognitive intervention in clinical practice, while also offering empirical data and clinical guidance.

## 2 Methods and materials

### 2.1 Study design and data source

The current cross-sectional research utilized case data derived from the hypertension electronic follow-up system of the Affiliated Hospital of Shandong University of Traditional Chinese Medicine. The system aggregated information from individuals with hypertension who received care in inpatient or outpatient settings at the Affiliated Hospital of Shandong University of Traditional Chinese Medicine, Jinan Hospital of Traditional Chinese Medicine, Jinan Minzu Hospital, Qingzhou People's Hospital, Penglai Hospital of Traditional Chinese Medicine, Tai'an First People's Hospital, Guangrao County Hospital of Traditional Chinese Medicine, Qufu Hospital of Traditional Chinese Medicine, and Weifang Hospital of Traditional Chinese Medicine between May 2022 and July 2024. Data were exported and consolidated into an Excel spreadsheet, yielding a total of 1,915 individuals. Inclusion criteria comprised: (i) age of 45–85 years; (ii) patients with essential hypertension; (iii) provision of signed informed consent; (iv) willingness to complete hypertension-related data collection and the assessment scales of anxiety, depression, and cognitive impairment. Exclusion criteria were: (i) patients with any form of secondary hypertension; (ii) vascular dementia or cognitive decline attributable to vascular factors; (iii) participation in a clinical trial of an investigational drug within the preceding three months; (iv) pregnant, pre-conception, or lactating women; (v) comorbid psychiatric disorders and/or psychoactive substance use disorder or dependence, and confirmed diagnosis with long-term anti-anxiety or antidepressant pharmacotherapy; (vi) malignant tumors or severely impaired hepatic, renal, or cardiac function; (vii) incomplete data.

### 2.2 Diagnostic criteria for hypertension

Hypertension was ascertained per at least one of the following criteria: (i) in the absence of antihypertensives, office systolic blood pressure (SBP) ≥ 140 mmHg and/or diastolic blood pressure (DBP) ≥ 90 mmHg on three separate occasions; (ii) current use of antihypertensives coupled with a documented history of hypertension, irrespective of blood pressure readings < 140/90 mmHg; (iii) mean SBP/DBP ≥ 130/80 mmHg via 24-hour ambulatory blood pressure monitoring; daytime mean ≥ 135/85 mmHg; nighttime mean ≥ 120/70 mmHg; (iv) in the absence of antihypertensives, home SBP/DBP ≥ 135/85 mmHg on three separate occasions (18).

### 2.3 Evaluation of CI

Cognitive function was evaluated through the Mini-Mental State Examination (MMSE). Normal cognitive function corresponded to an MMSE score of 27–30, whereas cognitive impairment was defined as an MMSE score <27 (19). The Chinese version of MMSE has demonstrated validity and reliability for assessing cognitive function within Chinese populations (20). The MMSE encompasses seven domains of simple tasks: temporal orientation, spatial orientation, immediate recall, attention and calculation, delayed recall, language ability, and visuospatial capacity. These comprise five sections: orientation (10 points), registration (3 points), attention and calculation (5 points), recall (3 points), and language (9 points). Total scores span 0 to 30; higher values indicate superior cognitive performance (21).

### 2.4 Evaluation of sleep quality

The Pittsburgh Sleep Quality Index (PSQI) was adopted to gauge sleep quality over the past month. The PSQI comprises 19 individual items across seven dimensions: subjective sleep quality, sleep latency, sleep duration, habitual sleep efficiency, sleep disturbances, use of sleep medication, and daytime dysfunction. Each item receives a score of 0–3. Aggregate PSQI scores span 0 to 21, with elevated scores signifying poorer sleep quality (22). A PSQI score > 5 conventionally indicates suboptimal self-reported sleep quality (23). Scores above 5 were categorized as fair sleep quality, and those ≤ 5 as good sleep quality.

### 2.5 Evaluation of anxiety and depressive symptom severity

Severity of anxiety symptoms in individuals with hypertension was appraised utilizing the Hamilton Anxiety Scale (HAMA), which consists of 14 items evaluated jointly by two raters. Higher scores reflect more pronounced anxiety: 0–7 denotes no or negligible anxiety; 8–14 indicates mild anxiety symptoms; 15–21 signifies definite anxiety of moderate intensity; > 22 implies severe anxiety markedly interfering with daily life and occupational functioning (24). An anxiety score > 7 denoted the presence of anxiety symptoms, while a score ≤ 7 indicated the absence of anxiety symptoms.

Severity of depressive symptoms was evaluated via the Hamilton Depression Scale (HAMD), comprising 24 items rated jointly by two trained evaluators. Higher scores denote greater depressive severity: a total score < 8 suggests absence of depressive symptoms; 8–20 points indicate possible mild depression; 20–35 confirms depression of moderate severity (higher scores denote greater severity); > 35 raises the possibility of severe depression (25). A depression score > 7 denoted the presence of depressive symptoms, while a score ≤ 7 indicated the absence of depressive symptoms.

### 2.6 Evaluation of physical activity

The long form of the International Physical Activity Questionnaire (IPAQ-L) represents an internationally validated instrument for appraising the levels of physical activity and sedentary behavior in adults. Developed by an expert panel, IPAQ-L exhibits robust reliability and validity and has undergone verification and applicability assessment across diverse cultural contexts, including China (26, 27). The IPAQ-L employs a retrospective self-report format to capture job-related, household, transportation, and leisure-time physical activities as well as sedentary time over the preceding seven days. Data are transformed into scores of metabolic equivalent tasks (METs) for each activity type and intensity. Weekly volume of physical activity (MET-minutes/week) is computed by multiplying total minutes per week for each activity by its designated MET value (walking: 3.3; moderate-intensity activity: 4; cycling: 6; vigorous activity: 8) and summing the resultant MET totals across all activities. Physical activity is grouped into three categories: (i) low (Category 1): lowest level of activity; criteria for Categories 2 or 3 are unmet; (ii) moderate (Category 2): meeting any of the following: ① ≥ 20 min of vigorous activity per day on ≥ 3 days; ② ≥ 30 min of moderate-intensity activity or walking per day on ≥ 5 days; ③ any combination of walking, moderate, or vigorous activity on ≥ 5 days per week achieving a cumulative total of at least 600 MET-minutes. (iii) high (Category 3): fulfilling either of the following: ① vigorous activity on ≥ 3 days per week accumulating at least 1,500 MET-minutes; ② a minimum cumulative total of 3,000 MET-minutes per week accrued through any combination of walking or moderate-to-vigorous activity over 7 consecutive days.

### 2.7 Study variables and grouping

Basic information was collected for all individuals with hypertension, encompassing educational attainment (low: illiteracy/elementary school; intermediate: junior/senior high school or equivalent; high: college/university or postgraduate), age, marital status, occupational type, sex, smoking, drinking, duration of hypertension, years since diagnosis of hypertension, family history (coronary heart disease, hypertension, diabetes mellitus, stroke, hyperlipidemia, malignancy), waist circumference (WC), height, hip circumference, weight, respiratory rate, heart rate, usual SBP, usual DBP, peak SBP/DBP (highest values self-reported by patients via questionnaire for the preceding week). Sleep-related parameters comprised: wake time, weekday sitting duration (minutes/day), weekend sitting duration (minutes/day), bedtime, sleep latency (minutes), frequency of difficulty initiating sleep, actual sleep duration (hours/day), weekday sleep duration (minutes/day), weekend sleep duration (minutes/day), and sleep quality. Clinical indices consisted of serum metabolic markers (total cholesterol, triglyceride, low-density lipoprotein cholesterol [LDL-C], high-density lipoprotein cholesterol [HDL-C], fasting blood glucose, serum creatinine) and echocardiographic parameters (left ventricular end-diastolic diameter, right ventricular end-diastolic diameter, left atrial diameter, right atrial diameter).

All subjects completed the assessments of HAMA, HAMD, and MMSE. Based on established scoring criteria, individuals were assigned to four groups: A (hypertension alone, *n* = 1,350) comprising patients without anxiety, depression, and/or cognitive impairment; B (hypertension with anxiety and depression, *n* = 309), comprising patients with concurrent anxiety and depressive symptoms; C (hypertension with anxiety, depression, and cognitive impairment, *n* = 65), comprising patients with coexisting anxiety, depression, and cognitive impairment; and D (hypertension with cognitive impairment, *n* = 191), comprising patients with cognitive impairment alone, without anxiety and/or depressive symptoms.

### 2.8 Statistical analysis

Statistical analyses were implemented in SPSS (v26.0). Normality of continuous data was examined via the Shapiro–Wilk test; all data exhibited non-normal distribution and were thus reported as median ± interquartile range. Intergroup comparisons for continuous data were carried out utilizing the Mann–Whitney U test for two groups and the Kruskal–Wallis one-way ANOVA for multiple groups. Categorical variables were expressed as frequencies/percentages and were compared via the chi-square test. Following differential analysis, variables demonstrating statistical significance were entered into binary logistic regression models—with reference groups specified according to the comparative analysis—to ascertain associated factors. Results were reported as odds ratios (ORs) with 95% confidence intervals (CIs) to quantify risk magnitude and direction. Receiver operating characteristic (ROC) curves were generated based on the identified associated indicators, and the area under the curve (AUC) with its corresponding 95% CI was derived. A two-tailed significance threshold of *P* < 0.05 was adopted.

## 3 Results

### 3.1 Baseline characteristics

Baseline characteristics of subjects are summarized in Tables 1, 2. Regarding demographics, individuals in Group B exhibited a significantly lower age relative to Group A (62 vs. 68 years), whereas the age of Group D did not differ from Group A and was markedly higher than that of Group B. The distribution of educational attainment varied across groups. Group B displayed the highest proportion of intermediate and high educational levels (72.2%), significantly exceeding that of Group A (57%). Educational profiles of Groups C and D resembled those of Group A. Additionally, Groups B and C demonstrated elevated proportions of individuals engaged in manual occupations (78.3% and 80.0%, respectively), whereas occupational distribution in Group D did not differ markedly from that of Group A.

**Table 1:** Basic information.

Variables		Total	A(hypertension alone)	B(Hypertension accompanied by anxiety and depression)	C(Hypertension accompanied by anxiety, depression and cognitive impairment)	D(Hypertension with cognitive impairment)	*H*/*χ^2^*	*P*
Age (years)		67 (60,74)	68 (60,74)	62 (58,70)^a^	67 (59,75)	68 (59,74)^b^	34.600	<0.001
Sex	Female	966 (50.4)	671 (49.7)	158 (51.1)	39 (60.0)	98 (51.3)	2.786	0.426
	Male	949 (49.6)	679 (50.3)	151 (48.9)	26 (40.0)	93 (48.7)		
Educational level	Low level	784 (40.9)	580 (43.0)	86 (27.8)^a^	29 (44.6)^b^	89 (46.6)^b^	41.292	<0.001
	Moderate level	1,045 (54.6)	724 (53.6)	194 (62.8)^a^	33 (50.8)	94 (49.2)^b^		
	High level	86 (4.50)	46 (3.40)	29 (9.40)^a^	3 (4.60)	8 (4.20)		
Marital status	With partner	1,869 (97.6)	1,322 (97.9)	301 (97.4)	62 (95.4)	184 (96.3)	3.961	0.235
	Without partner	46 (2.40)	28 (2.10)	8 (2.60)	3 (4.60)	7 (3.70)		
Job type	Physical labor	1,381 (72.1)	958 (71.0)	242 (78.3)	52 (80.0)	129 (67.5)^b^	13.919	0.031
	Mental work	222 (11.6)	162 (12.0)	33 (10.7)	6 (9.20)	21 (11.0)		
	Physical and mental work	312 (16.3)	230 (17.0)	34 (11.0)	7 (10.8)	41 (21.5)^b^		
Height		165 (160,172)	165 (160,173)	165 (160,170)^a^	165 (160,170)	165 (160,172)	11.067	0.011
Weight		70 (64,75)	70 (63,76)	70 (63,75)	70 (65,76)	70 (65,75)	0.311	0.958
Body mass index		25.07 (23.44,27.18)	24.9 (23.4,27.0)	25.7 (23.7,27.5)	26.2 (23.8,27.7)	25.1 (23.5,27.1)	7.695	0.053
Waistline^c^		89 (83,93)	88 (82,93)	90 (86,96)^a^	92 (87,96)^a^	88 (83,93)^b^^,^^c^	51.542	<0.001
Hip circumference		96 (92,102)	95 (91,101)	99 (95,108)^a^	98 (95,104)^a^	95 (90,101)^b^^,^^c^	72.172	<0.001
Breathe		18 (18,18)	18 (18,18)	18 (18,18)	18 (18,18)	18 (18,18)	1.560	0.668
Heart rate		74 (68,81)	72 (68,80)	76 (68,85)^a^	74 (68,80)	73 (68,80)	13.509	0.004
Smoking status	Never	1,379 (72.0)	940 (69.6)	252 (81.6)^a^	50 (76.9)	137 (71.7)	24.035	0.004
	Quit smoking	183 (9.60)	146 (10.8)	14 (4.50)^a^	6 (9.20)	17 (8.90)		
	Occasionally	57 (3.00)	45 (3.30)	3 (1.00)	1 (1.50)	8 (4.20)		
	Often	296 (15.5)	219 (16.2)	40 (12.9)	8 (12.3)	29 (15.2)		
Drinking status	Never	1,472 (76.9)	1,010 (74.8)	266 (86.1)^a^	55 (84.6)	141 (73.8)^b^	28.404	0.001
	Abstinence	87 (4.50)	60 (4.40)	14 (4.50)	2 (3.10)	11 (5.80)		
	Occasionally	191 (10.0)	153 (11.3)	10 (3.20)^a^	4 (6.20)	24 (12.6)^b^		
	Often	165 (8.60)	127 (9.40)	19 (6.10)	4 (6.20)	15 (7.90)		
Physical activity level	Low level	319 (16.7)	224 (16.6)	48 (15.5)	11 (16.9)	36 (18.8)	2.329	0.887
	Moderate level	976 (51.0)	699 (51.8)	154 (49.8)	32 (49.2)	91 (47.6)		
	High level	620 (32.4)	427 (31.6)	107 (34.6)	22 (33.8)	64 (33.5)		

Data are presented as *n* (%) and median (Q1, Q3). Differences were considered statistically significant at *P* < 0.05.

a denotes a statistical difference relative to Group A;.

b denotes a statistical difference relative to Group B;.

c denotes a statistical difference relative to Group C.

**Table 2:** Comparison of hypertension characteristics, family history, and treatment strategies.

Variables		Total	A(hypertension alone)	B(Hypertension accompanied by anxiety and depression)	C(Hypertension accompanied by anxiety, depression and cognitive impairment)	D(Hypertension with cognitive impairment)	*H*/*χ^2^*	*P*
Maximum systolic blood pressure		180 (160,180)	180 (160,185)	170 (150,180)^a^	175 (150,180)^a^	180 (160,185)^b^	49.587	<0.001
Maximum diastolic blood pressure		100 (100,110)	100 (100,110)	100 (90,100)^a^	100 (90,100)^a^^,^^b^	100 (100,110)^b^	75.989	<0.001
Systolic blood pressure at ordinary times		140 (130,150)	140 (130,150)	140 (130,150)^a^	140 (130,145)	140 (130,150)	13.615	0.003
Diastolic blood pressure at ordinary times		85 (80,90)	85 (80,90)	85 (80,90)	85 (80,90)	80 (80,90)	7.776	0.051
Course of disease		72 (36,138)	74 (37,141)	60 (31,129)	73 (26,140)	71 (34,132)	6.774	0.079
Years of hypertension		6 (3,11.5)	6.2 (3.1,11.8)	5.0 (2.6,10.8)	6.1 (2.2,11.7)	5.9 (2.8,11.0)	6.774	0.079
Age of hypertension		58.5 (51,65.17)	59.1 (51.3,65.8)	56.1 (49.3,62.0)^a^	59.4 (51.7,67.3)	57.6 (51.3,66.1)	19.321	<0.001
Age groups of hypertension	Young people	190 (9.9)	134 (9.90)	32 (10.4)	9 (13.8)	15 (7.90)	19.336	0.004
	Middle-aged adults	868 (45.3)	582 (43.1)	170 (55.0)^a^	25 (38.5)	91 (47.6)		
	Older adults	857 (44.8)	634 (47.0)	107 (34.6)^a^	31 (47.7)	85 (44.5)		
Family history of hypertension		1,053 (55.0)	843 (62.4)	69 (22.3)^a^	24 (36.9)^a^	117 (61.3)^b^^,^^c^	175.076	<0.001
Family history of hyperlipidemia		269 (14.0)	226 (16.7)	21 (6.80)^a^	5 (7.70)	17 (8.90)^a^	27.933	<0.001
Family history of coronary heart disease		466 (24.3)	371 (27.5)	40 (12.9)^a^	6 (9.20)^a^	49 (25.7)^b^^,^^c^	37.265	<0.001
Family history of stroke		277 (14.5)	228 (16.9)	17 (5.50)^a^	7 (10.8)	25 (13.1)^b^	27.486	<0.001
Family history of tumour		50 (2.60)	38 (2.80)	6 (1.90)	4 (6.20)	2 (1.00)	5.376	0.129
Family history of diabetes		230 (12.0)	181 (13.4)	19 (6.10)^a^	12 (18.5)^b^	18 (9.40)	16.308	0.001

Data are presented as *n* (%) and median (Q1, Q3). Differences were considered statistically significant at *P* < 0.05.

a denotes a statistical difference relative to Group A;.

b denotes a statistical difference relative to Group B;.

c denotes a statistical difference relative to Group C.

Concerning anthropometric measures, no marked differences emerged in height, weight, or body mass index among the groups. Nonetheless, WC (B: 90 cm; C: 92 cm vs. A: 88 cm) and hip circumference (B: 99 cm; C: 98 cm vs. A: 95 cm) were significantly greater in Groups B and C compared with Group A. In contrast, WC and hip circumference in Group D were notably smaller than those in Groups B and C. Resting heart rate in Group B was also significantly elevated relative to Group A (76 vs. 72 beats/min). With respect to lifestyle factors, the proportions of never-smokers (81.6% vs. 69.6%) and never-drinkers (86.1% vs. 74.8%) were markedly higher in Group B than in Group A. Group D diverged from Group B in terms of drinking, exhibiting a significantly greater proportion of occasional drinkers (12.6% vs. 3.2%). No marked disparities in the levels of physical activity were noted across groups (Table 1).

### 3.2 Comparison of blood pressure profiles and family history

For characteristics of blood pressure, both peak SBP and peak DBP were markedly lower in Groups B and C relative to Group A, whereas peak SBP in Group D was significantly higher than that in Group B. Intergroup differences in usual blood pressure were modest. Nonetheless, the age at the diagnosis of hypertension was notably younger in Group B than in Group A (*P* < 0.05), and Group B comprised a greater proportion of middle-aged individuals (55.0%) and a lower proportion of older adults (34.6%).

Regarding family history of disease, Groups B and C exhibited significantly lower rates of positive family history for hypertension, hyperlipidemia, coronary heart disease, and stroke relative to Group A. The family history profile of Group D more closely approximated that of Group A, and the rates of positive family history for hypertension and coronary heart disease in Group D were markedly higher than those in Groups B and C (*P* < 0.05; Table 2).

### 3.3 Investigation of clinical indices and sleep parameters

Metabolic and sleep indices were examined across the four clinical subtypes of hypertension. Fasting blood glucose and total cholesterol did not differ markedly among the groups (*P* > 0.05). Intergroup comparisons, however, revealed specific patterns of metabolic dysregulation. Relative to Group A, triglycerides, HDL-C, and LDL-C were all significantly reduced in Group B. This trend persisted for LDL-C in Group C. The levels of LDL-C in Group D were notably higher than those in Group C but did not differ statistically from Group A. Sleep profiles demonstrated that compared with Groups A and D, individuals in Groups B and C more frequently retired to bed after 22:00, exhibited prolonged sleep latency, experienced greater frequencies of difficulty initiating sleep, achieved lower PSQI scores, and reported poorer sleep quality (*P* < 0.001). Sleep latency, frequency of difficulty initiating sleep, PSQI score, and sleep quality in Group D did not differ significantly from those in Group A (*P* > 0.05). Relative to Group A, Group D displayed a tendency toward earlier wake times. In comparison with Group B, Group C exhibited longer weekday sitting durations (Table 3).

**Table 3:** Clinical metabolic and sleep-related indicators.

Variables		Total	A(hypertension alone)	B(Hypertension accompanied by anxiety and depression)	C(Hypertension accompanied by anxiety, depression and cognitive impairment)	D(Hypertension with cognitive impairment)	*H*/*χ^2^*	*P*
Fasting blood glucose		5.81 (5.17,7.17)	5.8 (5.2,7.2)	5.7 (5.1,7.1)	5.6 (5.1,7.6)	5.9 (5.0,7.5)	2.751	0.432
Triglyceride		1.31 (0.95,1.94)	1.3 (1.0,1.9)	1.4 (1.0,2.1)^a^	1.3 (0.9,2.0)	1.3 (0.9,2.1)	12.226	0.007
Cholesterol		4.6 (3.79,5.41)	4.6 (3.8,5.4)	4.6 (3.8,5.5)	4.3 (3.6,5.2)	4.7 (3.6,5.5)	2.852	0.415
HDL-C		1.18 (0.99,1.41)	1.2 (1.0,1.4)	1.1 (1.0,1.4)^a^	1.1 (1.0,1.3)	1.2 (1.0,1.4)	10.267	0.016
LDL-C		2.81 (2.14,3.48)	2.9 (2.2,3.6)	2.6 (2.0,3.2)^a^	2.4 (1.7,3.1)^a^	2.9 (2.1,3.6)^c^	35.541	<0.001
Serum creatinine		65.4 (55,78.1)	65.9 (56.0,78.6)	63.1 (53.0,75.5)^a^	63.0 (53.6,75.0)	67.0 (57.0,79.4)	10.413	0.015
Get-up time	Before 6: 00 am	873 (45.6)	650 (48.1)	127 (41.1)	24 (36.9)	72 (37.7)^a^	12.839	0.005
	After 6:00 am	1,042 (54.4)	700 (51.9)	182 (58.9)	41 (63.1)	119 (62.3)^a^		
Sitting hours per working day		220 (150,300)	240 (150,300)	200 (150,300)	250 (180,360)^b^	200 (120,300)	8.392	0.039
Sitting time on each rest day		240 (150,300)	240 (150,300)	200 (150,300)	300 (180,360)	200 (120,300)	7.202	0.066
Time to bed	Before 22:00 pm	1,269 (66.3)	972 (72.0)	144 (46.6)^a^	29 (44.6)^a^	124 (64.9)^b^^,^^c^	87.091	<0.001
	After 22:00 pm	646 (33.7)	378 (28.0)	165 (53.4)^a^	36 (55.4)^a^	67 (35.1)^b^^,^^c^		
Time to fall asleep (minutes)		20 (10,30)	20 (10,30)	30 (20,30)^a^	30 (20,60)^a^	15 (10,30)^b^^,^^c^	184.231	<0.001
Difficulty falling asleep	0	697 (36.4)	566 (41.9)	33 (10.7)^a^	9 (13.8)^a^	89 (46.6)^b^^,^^c^	217.407	<0.001
	Less than once	764 (39.9)	537 (39.8)	135 (43.7)	21 (32.3)	71 (37.2)		
	1–2 times	302 (15.8)	184 (13.6)	79 (25.6)^a^	19 (29.2)^a^	20 (10.5)^b^^,^^c^		
	More than 3 times	152 (7.90)	63 (4.70)	62 (20.1)^a^	16 (24.6)^a^	11 (5.80)^b^^,^^c^		
Actual sleep time		7 (7,8)	7 (7,8)	7 (7,7.5)^a^	7 (6,7.5)^a^	7 (6.5,8)	36.163	<0.001
Sleep time per working day		420 (400,480)	420 (400,480)	450 (420,480)^a^	470 (420,500)	450 (390,480)	11.733	0.008
Sleep time on each rest day		450 (400,480)	450 (400,480)	450 (420,500)^a^	480 (420,520)	450 (400,480)	13.336	0.004
PSQI		6 (4,8)	6 (4,7)	8 (6,10)^a^	9 (7,11)^a^	5 (3,7)^b^^,^^c^	250.848	<0.001
Sleep quality	Good	1,755 (91.6)	1,276 (94.5)	249 (80.6)^a^	48 (73.8)^a^	182 (95.3)^b^^,^^c^	94.147	<0.001
	General	160 (8.40)	74 (5.50)	60 (19.4)^a^	17 (26.2)^a^	9 (4.70)^b^^,^^c^		

Data are presented as *n* (%) and median (Q1, Q3). Differences were considered statistically significant at *P* < 0.05.

HDL-C, high-density lipoprotein cholesterol; LDL-C, low-density lipoprotein cholesterol; PSQI, Pittsburgh Sleep Quality Index.

a denotes a statistical difference relative to Group A;.

b denotes a statistical difference relative to Group B;.

c denotes a statistical difference relative to Group C.

### 3.4 Influence of antihypertensive use

The utilization rates of calcium channel blockers, angiotensin II receptor blockers (ARBs), *β*-blockers, and sympatholytic agents differed across the four groups (*P* < 0.05). No significant differences in the utilization rates of diuretics and angiotensin-converting enzyme inhibitors were observed among the groups (*P* > 0.05).

Pairwise comparisons further revealed that, relative to Group A, Groups B and C exhibited lower utilization rates of calcium channel blockers and ARBs, alongside higher utilization rates of sympatholytic agents (*P* < 0.05); the utilization rate of *β*-blockers in Group B was also lower than that in Group A (*P* < 0.05). Group D showed a significantly higher utilization rate of ARBs compared with Groups B and C, and a significantly higher utilization rate of *β*-blockers compared with Group B (all *P* < 0.05) (Supplementary Table S1).

Subsequent logistic regression demonstrated that, among patients with hypertension and cognitive impairment, only calcium channel blockers were associated with a significantly reduced risk of this comorbidity (OR=0.66, 95% CI: 0.47–0.91, *P* = 0.012). For hypertensive patients with comorbid anxiety and depression, calcium channel blockers, ARBs, and *β*-blockers were each linked to a significantly lower risk of anxiety and depression (all *P* < 0.001). Among individuals with concurrent anxiety, depression, and cognitive impairment, only ARBs were related to a significantly diminished risk of this combined condition (OR=0.46, 95% CI: 0.26–0.81, *P* = 0.007) (Supplementary Tables S2–S4).

### 3.5 Principal factors associated with hypertension comorbid with anxiety and depression

Based on differential analysis, binary logistic regression was carried out with hypertension plus anxiety and depression as the dependent variable, the hypertension-alone group as the reference category, and variables exhibiting marked disparities between Groups A and B as independent predictors. Results indicated that age (OR=0.94), height (0.96), lower peak SBP (0.98), lower peak DBP (0.97), occasional drinking (0.25), reduced frequency of family history of hypertension (0.15), and lower LDL-C (0.73) were protective factors against hypertension with anxiety and depression. Conversely, WC (1.06), heart rate (1.02), intermediate educational attainment (1.8), high educational attainment (2.51), alcohol cessation (3.1), usual SBP (1.02), bedtime after 22:00 (1.92), prolonged sleep latency (1.01), and fair sleep quality (4.04) emerged as independent risk factors. Notably, compared with individuals reporting no difficulty initiating sleep per week, increased frequency of difficulty initiating sleep exhibited a significant graded relationship with the odds of the outcome (ORs for < 1, 1–2, and ≥ 3 times were 3.99, 7.41, and 11.73, respectively; *P* < 0.05) (Table 4).

**Table 4:** Principal factors associated with hypertension and comorbid anxiety/depression.

Research variables	B	Se	Wald	*P*	OR (lower–upper)
Age	−0.063	0.011	30.231	<0.001	0.94 (0.92∼0.96)
Height	−0.042	0.013	9.942	0.002	0.96 (0.94∼0.98)
Waist circumference	0.059	0.009	38.971	<0.001	1.06 (1.04∼1.08)
Heart rate	0.016	0.007	5.611	0.018	1.02 (1.00∼1.03)
Educational attainment	-	-	8.69	0.013	-
Intermediate	0.589	0.216	7.454	0.006	1.8 (1.18∼2.75)
High	0.922	0.4	5.307	0.021	2.51 (1.15∼5.51)
Current drinking status (never)	-	-	21.494	<0.001	-
Former	1.131	0.455	6.194	0.013	3.10 (1.27∼7.55)
Occasional	−1.392	0.426	10.673	0.001	0.25 (0.11∼0.57)
Current	−0.422	0.373	1.284	0.257	0.66 (0.32∼1.36)
Peak systolic blood pressure	−0.02	0.006	11.473	0.001	0.98 (0.97∼0.99)
Peak diastolic blood pressure	−0.029	0.009	9.251	0.002	0.97 (0.95∼0.99)
Usual systolic blood pressure	0.024	0.007	10.131	0.001	1.02 (1.01∼1.04)
Family history of hypertension (Yes)	−1.87	0.188	98.447	<0.001	0.15 (0.11∼0.22)
LDL-C	−0.321	0.092	12.078	0.001	0.73 (0.61∼0.87)
Bedtime (after 22:00)	0.651	0.173	14.169	<0.001	1.92 (1.37∼2.69)
Sleep latency (minutes)	0.014	0.004	10.225	0.001	1.01 (1.01∼1.02)
Difficulty initiating sleep per week (never)	-	-	60.587	<0.001	-
< 1 time/week	1.384	0.235	34.684	<0.001	3.99 (2.52∼6.33)
1–2 times/week	2.002	0.278	52.001	<0.001	7.41 (4.30∼12.76)
≥ 3 times/week	2.462	0.393	39.297	<0.001	11.73 (5.43∼25.34)
Sleep quality (Fair)	1.396	0.327	18.183	<0.001	4.04 (2.13∼7.67)
Constant	4.662	2.724	2.93	0.087	-

LDL-C, low-density lipoprotein cholesterol; OR, odds ratio; 95% CI, 95% confidence interval. All odds ratios and confidence intervals were derived from logistic regression. *P* < 0.05 was considered statistically significant.

### 3.6 Principal factors associated with hypertension comorbid with anxiety, depression, and CI

Drawing upon univariate analysis, binary logistic regression was conducted with hypertension plus anxiety, depression, and CI as the dependent variable, the hypertension-plus-CI group as the reference, and variables demonstrating intergroup differences between Groups D and C as independent variables. The analysis identified WC (OR=1.07), sleep latency (1.06), and bedtime after 22:00 (3.12) as independent risk factors for hypertension with anxiety, depression, and CI. Increasing weekly frequency of difficulty initiating sleep displayed a dose-response relationship (ORs for < 1 time: 4.99; 1–2 times: 6.25; ≥ 3 times: 8.92). LDL-C (OR=0.62) represented a protective factor (Table 5).

**Table 5:** Principal factors associated with hypertension, anxiety, depression, and cognitive impairment.

Research variables	B	Se	Wald	*P*	OR (lower–upper)
Group D vs. Group C					
Waist circumference	0.066	0.02	10.691	0.001	1.07 (1.03∼1.11)
Family history of hypertension (Yes)	−0.931	0.387	5.787	0.016	0.39 (0.19∼0.84)
LDL-C	−0.48	0.202	5.65	0.017	0.62 (0.42∼0.92)
Bedtime (After 22:00)	1.134	0.38	8.908	0.003	3.12 (1.48∼6.55)
Sleep latency (minutes)	0.055	0.012	21.991	<0.001	1.06 (1.03∼1.08)
Difficulty initiating sleep per week (Never)	-	-	14.073	0.003	-
< 1 time/week	1.607	0.551	8.499	0.004	4.99 (1.69∼14.70)
1–2 times/week	1.832	0.58	9.986	0.002	6.25 (2.01∼19.46)
≥ 3 times/week	2.189	0.654	11.212	0.001	8.92 (2.48∼32.13)
Constant	−8.607	2.122	16.447	<0.001	-

LDL-C, low-density lipoprotein cholesterol; OR, odds ratio; 95% CI, 95% confidence interval. All odds ratios and confidence intervals were derived from logistic regression. *P* < 0.05 was considered statistically significant.

A subsequent binary logistic regression was conducted utilizing hypertension plus anxiety, depression, and CI as the dependent variable, the hypertension-alone group as the reference, and variables exhibiting differences between Groups A and D as predictors. Following adjustment for confounders, family history of hyperlipidemia (OR=0.49) emerged as a protective factor, whereas wake time before 06:00 (1.52) constituted a risk factor for this comorbid profile (Table 6).

**Table 6:** Principal factors associated with hypertension, anxiety, depression, and cognitive impairment.

Research variables	B	Se	Wald	*P*	OR (lower–upper)
Family history of hyperlipidemia (yes)	−0.708	0.265	7.150	0.007	0.49 (0.29∼0.83)
Wake time (before 06:00)	0.418	0.159	6.884	0.009	1.52 (1.11∼2.08)
Constant	−2.106	0.127	273.722	<0.001	-

OR, odds ratio; 95% CI, 95% confidence interval. All odds ratios and confidence intervals were derived from logistic regression. *P* < 0.05 was considered statistically significant.

An additional binary logistic regression was carried out using hypertension plus anxiety, depression, and CI as the dependent variable, the hypertension-plus-anxiety-and-depression group as the reference, and variables displaying differences between Groups B and C as predictors. Family history of diabetes mellitus (OR=3.06) was identified as a risk factor (Table 7).

**Table 7:** Principal factors associated with hypertension, anxiety, depression, and cognitive impairment.

Research variables	B	Se	Wald	*P*	OR (lower–upper)
Family history of diabetes mellitus (yes)	1.117	0.405	7.613	0.006	3.06 (1.38∼6.76)
Weekday sitting duration	0.003	0.001	5.707	0.017	1.00 (1.00∼1.01)
Constant	−2.384	0.336	50.188	<0.001	-

OR, odds ratio; 95% CI, 95% confidence interval. All odds ratios and confidence intervals were derived from logistic regression. *P* < 0.05 was considered statistically significant.

### 3.7 Clinical utility of associated factors

**Figure 1:**
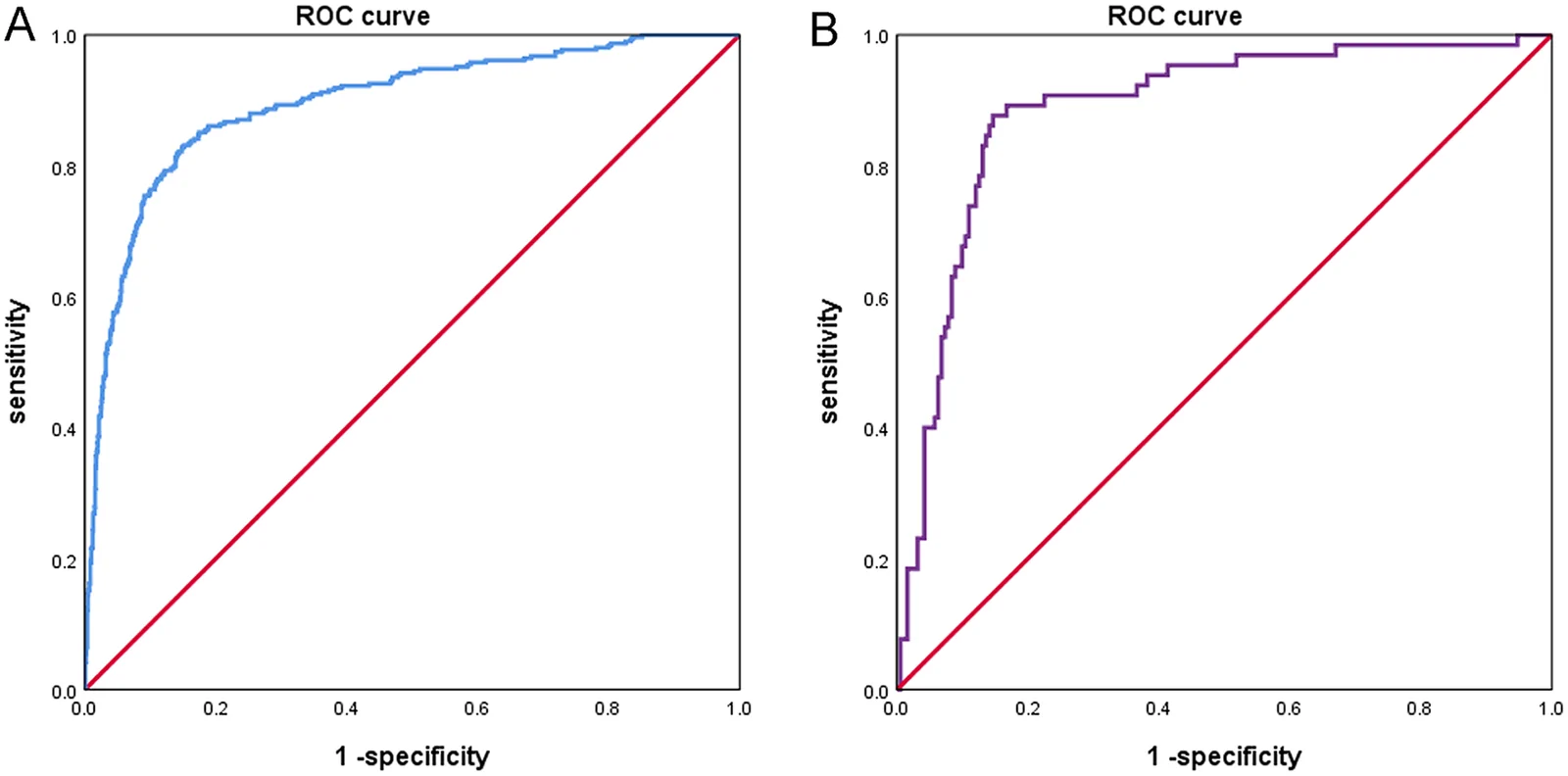
ROC curves. **(A)** Hypertension with anxiety and depression; **(B)** Hypertension with anxiety, depression, and cognitive impairment.

ROC curve analysis (Figure 1A) demonstrated that, with the hypertension-alone group serving as the reference, the factors linked to hypertension with anxiety and depression exhibited robust discriminative capacity [AUC: 0.895 (95% CI: 0.874–0.916)], yielding a sensitivity of 83.2% and a specificity of 84.8%. ROC analysis (Figure 1B) further indicated that, relative to the hypertension-with-CI reference, the factors associated with hypertension plus anxiety, depression, and CI possessed excellent discriminatory performance [AUC: 0.887 (95% CI: 0.839–0.934)], achieving a sensitivity of 87.7% and a specificity of 85.3%.

## 4 Discussion

Through fine-grained phenotyping of 1,915 middle-aged and older hypertensive patients, the present investigation systematically delineated pivotal factors associated with anxiety and depressive symptoms and their comorbidity with cognitive impairment. The findings indicate that the proportion of hypertensive patients with comorbid anxiety and depressive symptoms was 16.14%, exceeding the reported prevalence of this comorbidity in the general population (9.0%) (28, 29). This discrepancy is likely attributable to differences in population selection. Elevated arterial blood pressure constitutes a principal cause of cognitive impairment, and the prevalence of cognitive impairment rises with advancing age (30); the current results align with this pattern. The prevalence of hypertension with concomitant anxiety, depression, and cognitive impairment has seldom been documented in prior research; this investigation yielded an estimate of approximately 3.4%.

The current analysis revealed that hypertensive patients with comorbid anxiety and depression exhibited a younger age at first onset, with middle-aged patients accounting for a higher proportion, followed by older patients. This finding is consistent with earlier reports (31). Patients with hypertension and comorbid anxiety and depression displayed lower peak blood pressure, a result that is not fully consistent with available evidence concerning the association of blood pressure levels with anxiety and depression. One investigation reports a marked negative association between depression and both systolic and diastolic blood pressure (32); however, a six-year cohort study detects no link between hypertension and depression (33). Other research has documented a marked positive association of anxiety comorbidity with hypertension (34). A Finnish study demonstrates that individuals with high levels of anxiety have higher mean systolic and diastolic blood pressure relative to those with low levels of anxiety. It is postulated that anxiety may elevate baseline blood pressure and augment variability in blood pressure by activating the sympathetic nervous system (35). Potential sources of these discrepancies include differences in study methodology, selection of key variables, analytical approaches, and population characteristics.

A family history of hypertension is an established risk factor for the development of hypertension (36, 37). The present investigation found that hypertensive patients with a family history of hypertension were more susceptible to anxiety and depression. This finding is corroborated by Karacan et al., who find that a family history of hypertension among close relatives heightens health awareness and concern regarding the condition, precipitating marked changes in anxiety and attitudes (38). A cross-sectional study from Ethiopia indicates that hypertensive patients without a family history of hypertension have a 61% lower likelihood of depression. This pattern is possibly explained by the absence of genetic interaction between depression and hypertension within families (39).

In the current investigation, WC and heart rate were independent risk factors for comorbid anxiety and depression in patients with hypertension. Prior evidence has indicated a high degree of comorbidity linking obesity and WC to anxiety and depression. WC is, in turn, closely associated with hypertension and metabolic disturbances (40). Anxiety has been shown to reduce heart rate variability (HRV) and elevate blood pressure, and diminished HRV itself serves as a mediating mechanism in the anxiety–blood pressure association (41). Hypertensive patients with a higher educational attainment exhibited an elevated risk of anxiety and depression, contrasting sharply with prior research that identified low educational level as a risk factor (42). Higher education levels can enhance health literacy. However, individuals with higher education levels may be more likely to experience feelings of frustration and loss of control after diagnosis of chronic diseases due to higher social expectations and occupational pressures. At the same time, those with higher education levels have a deeper understanding of the long-term complications of hypertension, which may lead to excessive worry about future health outcomes. Hypertension patients with higher education levels are more prone to anxiety and depression, which may be related to high intelligence. Further research is needed to clarify our findings (43).

In terms of lifestyle habits, our findings indicated that former drinkers had an approximately 2.1-fold elevated risk of anxiety and depression relative to individuals who never drank. This observation aligns with the ’sick quitter effect’, which posits that the elevated risk is attributable to poor health or pre-existing psychological distress compelling individuals to quit drinking, rather than to the act of abstinence itself. Prior investigations have suggested that former drinker populations include many individuals who ceased drinking because of worsening disease, and their health status is inherently poorer (44). Katikireddi et al. similarly documented a comparable ‘reverse causation’ phenomenon in a large cohort study, wherein poor health may precede the behavior of alcohol cessation (45). Furthermore, studies have indicated significant differences in health status between heavy drinkers and lifelong abstainers, with the former often carrying a greater burden of chronic conditions, and the disease burden itself may be a risk factor for anxiety and depression (46).

The present investigation found that sleep onset before 22:00 was associated with greater variability in longitudinal systolic blood pressure compared with sleep onset between 22:00 and midnight (47). A large population-based study conducted in rural China demonstrates that PSQI components such as poor sleep quality and prolonged sleep latency are associated with hypertension (48). Another cross-sectional investigation reveals a synergistic effect of sleep difficulties and depression on hypertension (49). The current analysis found that sleep disturbances exerted a marked influence on hypertensive patients with comorbid anxiety and depression and on those with comorbid anxiety, depression, and cognitive impairment, whereas no association was detected with cognitive impairment alone. Accordingly, it is inferred that the impact of sleep disturbances may be linked to affective disorders *per se*. Maladaptive sleep behaviors may induce sustained increases in sympathetic tone through nocturnal sympathetic overactivation, subsequently elevating blood pressure (50).

Of particular note, lower concentrations of LDL may serve as a protective factor for hypertensive patients with comorbid anxiety and depression and for those with comorbid anxiety, depression, and cognitive impairment. This pattern is intimately related to the complex interplay among lipid metabolism, cardiovascular health, and neuropsychiatric disorders. The protective role of low LDL levels in hypertension is widely acknowledged: low LDL-C reduces the risk of cardiovascular events by attenuating atherosclerosis (51). Hypertensive patients generally exhibit elevated prevalence rates of anxiety and depression; research has identified a link between lipid parameters and depression, with inflammation possibly exerting a mediating effect (52). Optimizing cardiovascular health through improved lipid profiles may indirectly ameliorate mood disturbances. Furthermore, evidence indicates that hypercholesterolemia constitutes an early risk factor for cognitive decline, and maintaining lower concentrations of LDL-C may protect cognitive function by mitigating neuroinflammation and improving the integrity of the blood–brain barrier (53).

### 4.1 Limitations and future directions

Several limitations merit acknowledgment. The temporal sequence of causal relationships among variables cannot be established due to the cross-sectional design. Although patients with certain severe conditions were excluded, selection bias may still exist in the sample. Moreover, the differential impact of varying severity levels of anxiety and depression on cognitive function was not thoroughly examined. Future cohort studies or interventional trials are required to verify the validity and generalizability of these findings.

## 5 Conclusion

This research delineated critical risk and protective factors associated with anxiety and depressive symptoms combined with CI in individuals with hypertension. For individuals with hypertension who also present with anxiety and depression or comorbid CI, priority should be accorded to interventions targeting sleep (e.g., alleviating difficulty initiating sleep, adjusting bedtime to before 22:00), management of WC, and prudent regulation of LDL-C levels. The diagnostic model established herein furnishes a convenient tool for early identifying individuals with hypertension at elevated risk for anxiety, depression, and concomitant CI, thereby facilitating optimization of emotional management and cognitive intervention strategies. Through its multicenter cross-sectional design, the present research addresses a research gap concerning the influence of comorbid hypertension and anxiety/depression on cognitive function, clarifies pivotal associated factors such as sleep disturbance, and supplies new targets and empirical support for mechanistic exploration and clinical management of this complex comorbidity.

## Data Availability

The original contributions presented in the study are included in the article/Supplementary Material, further inquiries can be directed to the corresponding author.

## Supplementary material

Supplementary Table S1 Antihypertensive use among individuals with hypertension.

Supplementary Table S2 Influence of antihypertensive use on hypertension with cognitive impairment.

Supplementary Table S3 Influence of antihypertensive use on hypertension with anxiety and depression.

Supplementary Table S4 Influence of antihypertensive use on hypertension with anxiety, depression, and cognitive impairment.
