# Supplementary material for: Factors associated with anxiety, depression, and cognitive impairment in individuals with hypertension: a cross-sectional study based on a Chinese population
Source: Front Cardiovasc Med. 2026 Aug 11;13:1888483. doi: 10.3389/fcvm.2026.1888483 (PMC13504679; doi:10.3389/fcvm.2026.1888483)
Supplement: Supplementary file 1 [file Table1.docx]

**Supplementary Materials**

**Supplementary Table S1** Antihypertensive use among individuals with hypertension.

|  |  | A | B | C | D | Statistic | P |
| --- | --- | --- | --- | --- | --- | --- | --- |
| Calcium channel blockers | No | 571(42.3) | 184(59.5)^a^ | 39(60.0)^a^ | 97(50.8) | 36.901 | 0.000 |
|  | Yes | 779(57.7) | 125(40.5)^a^ | 26(40.0)^a^ | 94(49.2) |  |  |
| Diuretics | No | 1197(88.7) | 287(92.9) | 58(89.2) | 173(90.6) | 5.012 | 0.171 |
|  | Yes | 153(11.3) | 22(7.10) | 7(10.8) | 18(9.40) |  |  |
| Angiotensin II receptor blockers | No | 483(35.8) | 189(61.2)^a^ | 37(56.9)^a^ | 72(37.7)^bc^ | 74.922 | 0.000 |
|  | Yes | 867(64.2) | 120(38.8)^a^ | 28(43.1)^a^ | 119(62.3)^bc^ |  |  |
| Angiotensin-converting enzyme inhibitors | No | 1333(98.7) | 305(98.7) | 64(98.5) | 187(97.9) | 1.465 | 0.641 |
|  | Yes | 17(1.30) | 4(1.30) | 1(1.50) | 4(2.10) |  |  |
| β-blockers | No | 1021(75.6) | 268(86.7)^a^ | 54(83.1) | 138(72.3)^b^ | 21.845 | 0.000 |
|  | Yes | 329(24.4) | 41(13.3)^a^ | 11(16.9) | 53(27.7)^b^ |  |  |
| Sympatholytic agents | No | 1342(99.4) | 300(97.1)^a^ | 64(98.5) | 190(99.5) | 11.580 | 0.005 |
|  | Yes | 8(0.60) | 9(2.90)^a^ | 1(1.50) | 1(0.50) |  |  |

Note: ^a^ denotes a statistical difference relative to Group A; ^b^ denotes a statistical difference relative to Group B; ^c^ denotes a statistical difference relative to Group C.

OR stands for odds ratio, and 95% CI is the 95% confidence interval. All ORs and confidence intervals were obtained through logistic regression analysis, and P < 0.05 was considered statistically significant.

**Supplementary Table S2** Influence of antihypertensive use on hypertension with cognitive impairment.

|  | B | Se | Wald | P | OR (lower limit–upper limit) |
| --- | --- | --- | --- | --- | --- |
| Group A vs. Group D |  |  |  |  |  |
| Calcium channel blockers | -0.42 | 0.168 | 6.299 | 0.012 | 0.66 (0.47-0.91) |
| Diuretics | -0.286 | 0.267 | 1.148 | 0.284 | 0.75 (0.45-1.27) |
| Angiotensin II receptor blockers | -0.211 | 0.175 | 1.464 | 0.226 | 0.81 (0.58-1.14) |
| Angiotensin-converting enzyme inhibitors | 0.305 | 0.577 | 0.279 | 0.597 | 1.36 (0.44-4.20) |
| β-blockers | 0.178 | 0.176 | 1.022 | 0.312 | 1.20 (0.85-1.69) |
| Sympatholytic agents | -0.298 | 1.075 | 0.077 | 0.781 | 0.74 (0.09-6.10) |
| Constant | -1.617 | 0.189 | 73.368 | <0.001 | 0.198 |

Note: OR represents the odds ratio, and 95%CI is the 95% confidence interval; all ORs and confidence intervals are obtained through logistic regression analysis, and P<0.05 is considered to be statistically significant.

**Supplementary Table S3** Influence of antihypertensive use on hypertension with anxiety and depression.

|  | B | Se | Wald | P | OR (lower limit–upper limit) |
| --- | --- | --- | --- | --- | --- |
| Group A vs. Group B |  |  |  |  |  |
| Calcium channel blockers | -1.021 | 0.14 | 52.92 | <0.001 | 0.36 (0.27-0.47) |
| Angiotensin II receptor blockers | -1.263 | 0.14 | 81.216 | <0.001 | 0.28 (0.21-0.37) |
| β-blockers | -0.688 | 0.185 | 13.822 | <0.001 | 0.50 (0.35-0.72) |
| Sympatholytic agents | 0.988 | 0.532 | 3.455 | 0.063 | 2.69 (0.95-7.62) |
| Constant | -0.208 | 0.125 | 2.76 | 0.097 | 0.812 |

Note: OR represents the odds ratio, and 95%CI is the 95% confidence interval; all ORs and confidence intervals are obtained through logistic regression analysis, and P<0.05 is considered to be statistically significant.

**Supplementary Table S4** Influence of antihypertensive use on hypertension with anxiety, depression, and cognitive impairment.

|  | B | Se | Wald | P | OR (lower limit–upper limit) |
| --- | --- | --- | --- | --- | --- |
| Group A vs. Group C |  |  |  |  |  |
| Angiotensin II receptor blockers | -0.781 | 0.292 | 7.176 | 0.007 | 0.46 (0.26-081) |
| Constant | -0.666 | 0.202 | 10.832 | 0.001 | 0.51 |

Note: OR represents the odds ratio, and 95%CI is the 95% confidence interval; all ORs and confidence intervals are obtained through logistic regression analysis, and P<0.05 is considered to be statistically significant.
